# Acute effects of a single electroacupuncture session on purinergic signaling and inflammatory markers in patients with chronic nonspecific low back pain: a pilot study

**DOI:** 10.1007/s11302-026-10163-5

**Published:** 2026-05-14

**Authors:** Jardel Cristiano Ecco, Vinicius Ansolin, Adinei Abadio Soares, Angela Makeli Kososki Dalagnol, Filomena Marafon, Francini Franscescon, Matheus Chimelo Bianchini, Débora Tavares de Resende e Silva

**Affiliations:** 1 Laboratory of Genetic and Biochemistry, Federal University of Fronteira Sul (UFFS), SC 484 Highway, Southern Border, Chapecó, SC 89815-899 Brazil; 2 Graduate Program in Biomedical Sciences, Federal University of Fronteira Sul (UFFS), Rodovia SC 484 - Km 02, Fronteira Sul, SC 484 Highway, Southern Border, Chapecó, SC 89815-899 Brazil

**Keywords:** Adenosine, ATP, Interleukins, IL-10, Ectonucleoside

## Abstract

Chronic nonspecific low back pain (CNLBP) is a problem faced by a significant portion of the world population. However, among the strategies for treating these patients, there are pharmacological and nonpharmacological options. However, noninvasive therapies are options that have been gaining prominence. Among them, electroacupuncture (EA) stands out, which can be used to treat this type of pain. Thus, the objective of our study is to investigate whether only one session with an EA protocol in patients with NCLBP could modulate the purinergic system and the levels of inflammatory cytokines. For this study, a total of 23 patients with CNLBP, of both sexes, were treated and underwent an EA session lasting 20 min. The EA protocol was performed with a needling depth of 5 mm at the acupoints (BL23, bilateral), (BL25, bilateral), (BL40, bilateral), (SP6, bilateral) and (GB-39, bilateral) with intermittent waves, low frequency of 2 Hz and pulse width of 10 mA. Blood samples were collected for analysis before and after the EA protocol. Our results, after only one session, showed a significant increase in AMP hydrolysis by ecto-5'-nucleotidase/CD73 enzyme (*p* = 0.0002), adenosine deaminase activity (*p* = 0.0382) and extracellular ATP levels (*p* = 0.0036). Furthermore, the EA protocol increased the expression of NTPDase/CD39 (*p* = 0.0287) and ecto-5'-nucleotidase/CD73 (*p* = 0.0007) after the 20-min treatment. Furthermore, regarding inflammatory markers, there was a significant decrease in INF-γ (*p* = 0.0192), IL-4 (*p* = 0.0011) and IL-6 (*p* = 0.0156) associated with an increase in IL-10 (*p* = 0.0031). Moreover, after one EA session increased of the CD73 expression and adenosine deaminase were strong negative correlated with inflammatory cytokines TNF-α and IL-6, respectively. The upregulation of the purinergic system, likely associated with an indirect increase in adenosine levels, may promote a shift toward an anti-inflammatory profile, characterized by increased anti-inflammatory interleukins and reduced pro-inflammatory cytokines. Thus, our results demonstrate that the beneficial effects associated with EA can be evidenced from its first session due to the ability of this therapy to modulate the purinergic system and directly or indirectly modify the levels of pro- and anti-inflammatory interleukins.

**Graphical Abstract:**

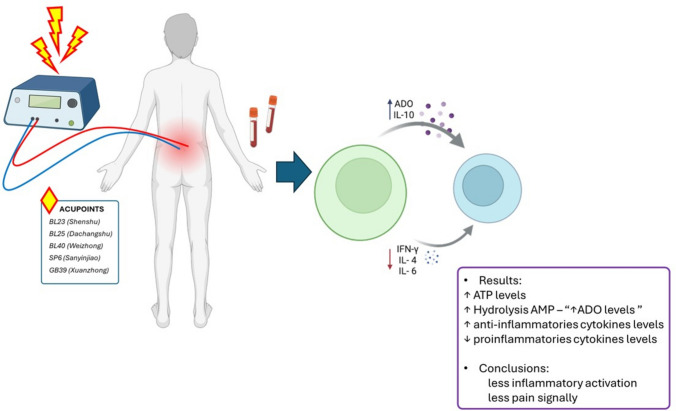

## Introduction

Chronic nonspecific low back pain (CNLBP) is defined as pain or discomfort localized between the lower edge of the ribs and the buttock. Occurring for more than three months that is not attributable to a specific pathology (*e.g*. infection, tumor, osteoporosis, lumbar spine fracture, structural deformity, inflammatory disorder, radicular syndrome, or cauda equina syndrome [1]. The duration of pain can last for a short time (acute/under 6 weeks), a little longer (sub-acute/6–12 weeks) or a long time (chronic/over 12 weeks) and can affect anyone. Additionally, the highest number of cases of CNLBP occurs between the ages of 50 and 55, with a higher prevalence in women [2]. In 2020, CNLBP affected around 619 million people worldwide and it is estimated that the number of cases will increase to 843 million cases by 2050 [3]. Common symptoms include lumbar stiffness, especially after rest or inactivity, and difficulty with basic movements like bending or lifting. Muscle spasms are frequent and intensify pain, further restricting mobility. In some cases, the pain radiates to the buttocks, thighs, or legs, indicating possible nerve root compression, as in sciatica. Additional symptoms may include tingling, numbness, or, in more severe cases, muscle weakness in the lower limbs [4]. The symptoms and signs of CNLBP, from onset to persistent pain, may be associated with different molecular alterations in patients. Among the mechanisms involved are intermediaries such as cytokines and inflammatory mediators, which are critical components of the immune response triggered by injuries or harmful stimuli [5]. Beyond their immunological functions, pro-inflammatory cytokines contribute to pain by directly sensitizing nociceptors or by promoting the release of substances that influence nociceptive signaling at both peripheral and central levels [6].

CNLBP is a major cause of disability, absenteeism, and high medical expenses. Adequate treatment of CNLBP is therefore important for patients, clinicians, and healthcare policy makers. Alternative uses to CNLBP are educating patients to prevent new events, pharmacological (*e.g.* anti-inflammatories and antidepressive) and nonpharmacological (e.g. physical exercise and physiotherapy) strategies to avoid the pain to patients [7]. However, chronic LBP often requires a long time of treatment or does not always respond to conventional therapies such as exercise or medicine [1]. In 2017, the American College of Physicians (ACP) published clinical guidelines outlining noninvasive approaches for managing acute, subacute, and chronic low back pain. For chronic CNLBP specifically, the guidelines emphasize that nonpharmacologic therapies should be considered as the initial treatment option [8]. One alternative to use in patients with CNLBP noninvasive is electroacupuncture (EA), a traditional Chinese medicine technique combining acupuncture with electrical stimulation [9]. Mechanism of action of the EA can be a multitarget characteristic of therapy. The analgesic and anti-inflammatory effects of EA are complex processes that involve multiple levels, pathways, and targets. These effects engage both peripheral and central mechanisms, including the opioid peptide system, the endocannabinoid system, purinergic signaling, the monocyte-macrophage system, and glial cells distributed within these networks. Various inflammatory factors and mediators are also closely associated [10]. Research has shown that analgesia of EA effect is essentially a manifestation of integrative processes at different levels in the central and peripheral nervous system involving neurotransmitters and neuromodulators [11].

Neural and immunological networks play a crucial role in the mechanisms underlying the effects of EA [12]. In the nervous system, EA primarily exerts analgesic effects, while in the immune system, it promotes anti-inflammatory actions. However, the interaction between these two systems is fundamental for achieving the full therapeutic effects of EA [13]. Among the mechanisms involved, pro- and anti-inflammatory cytokines are essential for the initiation, perpetuation, and regulation of the immune response [10]. In addition to their role in the immune response, proinflammatory cytokines also sensitize nociceptors either directly or indirectly through agents that modulate nociception at the peripheral or central level [14]. Previous studies have demonstrated significantly elevated levels of tumor necrosis factor-alpha (TNF-α), interleukin-1 beta (IL-1β), and interleukin-6 (IL-6) in individuals with acute and chronic pain [15, 16]. Purinergic signaling represents one of the key pathways involved in the initiation of acupuncture effects [11]. Purinergic signaling is present in different cells of the body and plays important roles in regulating immune activation, inflammation and pain [17]. Purinergic receptors (P1—A_1_, A_2A_, A_2B_, A_3_) are activated by adenosine (ADO) whereas P2 receptors (P2X_1_—P2X_7_) and (P2Y_1,2,4,6,11–14_) are stimulated by adenosine triphosphate (ATP), adenosine diphosphate (ADP) and uridine triphosphate (UTP), uridine diphosphate (UDP) and UDP-glucose [18, 19]. Purines such as ATP and ADP are hydrolyzed at AMP by ectonucleoside triphosphate diphosphohydrolase 1 (ENTPD1/CD39). AMP is hydrolyzed in adenosine by ecto-5'-nucleotidase (e5NT/CD73), and adenosine deaminase (ADA) converts adenosine in inosine [20]. Purinergic signaling can trigger both pro-inflammatory and anti-inflammatory responses and is implicated in the modulation of nociception and antinociception [21]. Previous studies have highlighted the involvement of purinergic pathways as a fundamental cellular mechanism underlying the analgesia induced by EA [22]. Adenosine released after acupuncture has anti-nociceptive action through A1 receptor [22]. In addition, EA may stimulate analgesia mainly through conversion of ATP in adenosine [23]. However, although numerous studies have investigated the therapeutic effects of EA in chronic low back pain, studies addressing these effects from the perspective of purinergic signaling remain limited.

Considering the impact that low back pain can have on individuals' quality of life and the need for a deeper mechanistic understanding of how non-pharmacological treatments may act. The aim of our study was to evaluate whether a single/acute session of EA can modulate the inflammatory response via the purinergic system in patients with chronic nonspecific low back pain. Our hypothesis is that EA alleviates nociception in these patients by modulating the systemic inflammatory response. The results observed after a single session of EA included changes in the activity and expression of enzymes involved in immune cell communication, as well as a reduction in pro-inflammatory interleukins and an increase in anti-inflammatory interleukins in patients with CNLBP.

## Materials and methods

### Experimental design

This was a quasi-experimental before-and-after study in patients with CNLBP. This research is part of an umbrella project entitled “The impact of non-pharmacological practices, such as physical exercises and integrative complementary practice as alternative treatments for chronic pain in chronic health conditions”, which was approved by the Human Research Ethics Committee of UFFS—number CAAE Nº 70754123.0.0000.5564.

### Population and sampling

The sample size calculation was performed using the GPower 8.1.1 program (Heinrich Heine Universität Düsseldorf, Germany), in which α = 0.05 and power of 80%. The total sample size obtained was 19 participants. Considering the possibility of sample loss of 20%, as a safety margin, it was decided to collect a total number of 23 individuals.

The inclusion criteria were reporting pain in the area between the 12th rib and the gluteal fold, which could be accompanied by pain radiating to the leg with a back pain intensity of at least 2 on a numerical rating scale from 0 to 10; CNLBP for more than 3 months; and being between 18 and 65 years of age [24, 25]. The main exclusion criteria were a positive response to the straight leg raise test and the Slump test, which have good diagnostic accuracy for lumbar radiculopathy [26, 27], no previous self-reported diagnosis of spinal stenosis, fracture, malignant tumor of any type, degenerative, infectious or rheumatic disease (rheumatoid arthritis, ankylosing spondylitis and osteoarthritis), or previous surgery on the lumbar spine [28, 29].

Participants were allowed to use analgesics and anti-inflammatories, if they stopped five days before blood collection. In addition, volunteers were instructed to stop consuming caffeine 12 h before testing [30]. All volunteers were informed about the experimental procedures before giving their written consent and were also informed that they could end their participation at any time. During the development of the research, the collected material remained in the possession of the researcher in charge and in the freezer in the research laboratory on the premises of UFFS, Chapecó Campus, to which only the researchers involved have access.

### Intervention protocols

The procedure was performed in a single EA session to verify the acute effects before and 30 min after the intervention, carried out in a suitable room at UFFS, Chapecó—SC. At baseline, participants completed questionnaires on demographic information: age, sex, comorbidities, duration of pain, whether they had previously had episodes of low back pain, education, whether they were undergoing any treatment, whether they were using any medication, whether they practiced physical activity.

Pain intensity was assessed at baseline using the Numeric Pain Rating Scale (NPRS), where 0 represents no pain and 10 represents the worst pain imaginable. The NPRS has been shown to have a high interobserver reliability coefficient (r = 0.88) [31, 32]. Disability due to pain was assessed using the Roland Morris Disability Questionnaire (RMDQ), a short and simple measure with proven validity, reliability, and responsiveness. The questionnaire is a 24-item scale, with scores ranging from 0 (no disability) to 24 (high disability). The RMDQ has been successfully translated and adapted for use in Brazilian patients, with satisfactory reliability and cross-sectional validity [33]. Participants were comfortably positioned in the prone position and received 20 min of electrical stimulation after all needles were placed [25].

The acupuncture points that were used followed the protocol by [34]. The acupoints were BL23 (Shenshu), BL25 (Dachangshu), BL40 (Weizhong), SP6 (Sanyinjiao) and KI3 (Taixi). EA was performed with a needling depth of 5 mm, with 0.25 × 30 m needles. The EA stimulation parameters were intermittent wave, low frequency of 2 Hz and pulse width of 10 mA. The stimulation intensity was adjusted once to reach a comfortable level during treatment. The EA equipment used in the study was the Novo EL608 Digital Connect electro stimulator.

### Biological material collection

Blood samples were collected using tubes of 10 mL with ethylenediaminetetraacetic acid (EDTA) or separator gel and clot activator. Blood was collected before starting and after the end of resistance exercise protocol. The samples were processed and fractionated into by-products (plasma, platelets, and leukocytes). The samples were processed and separated by centrifugation (5000 rpm, for 15 min, 20 ºC). Biologic samples were stored in microtubes of 1.5 mL at −80 °C for further analysis.

### Isolation of mononuclear cells from human blood

The buffy coat containing the white blood cells, placed between the layer of plasma and erythrocytes, was carefully removed. The material was placed in a conical tube and diluted in an equal volume of saline solution. Subsequently, the diluted sample was transferred to a conical tube containing Lymphoprep (Ficoll-Histopaque), followed by centrifugation at 1800 revolutions per minute (rpm), at room temperature, for 30 min. After centrifugation, a density gradient was formed. In this way, there was the formation of an intermediate layer composed of mononuclear cells (lymphocytes and monocytes) between the layers of plasma and Ficoll. This cloud of cells was carefully removed with a Pasteur pipette over the upper layer (plasma) and transferred to a clean conical tube. Over the cells, 10 ml of saline solution was added followed by centrifugation for 10 min at 1500 rpm. Then, we discarded the supernatant and added 5 ml of saline solution to completely wash the cells and remove the platelets. If necessary, EDTA-Ammonium Chloride hemolytic buffer can be added to remove red blood cells. Thus, a lymphocyte concentrate was obtained. Mononuclear cells were employed in further analysis.

### Analysis of the purinergic signaling

#### E-NTPDase enzyme assays

After lymphocyte isolation, E-NTPDase activity was determined as described [35]. The reaction medium contained 0.5 mmol/l CaCl2, 120 mmol/l NaCl, 5 mmol/l KCl, 6 mmol/l glucose and 50 mmol/l Tris–HCl buffer at pH 8.0, with a final volume of 160 μl. 20 µl of the intact mononuclear cells suspended in saline solution was added to the reaction medium (0.1 a 0.2 mg/mL of protein) and pre-incubated for 10 min at 37 °C and after the first incubation, 20ul of ATP or ADP nucleotides was added and a new incubation of 70 min at 37 °C was done. The reaction was initiated by the addition of substrate (ATP or ADP) at a final concentration of 2.0 mmol/l and stopped with 150 μl of 15% trichloroacetic acid (TCA). After suspending the reaction with the addition of TCA, add 200ul of the colorimetric reagent to 20ul of the final product. The released inorganic phosphate (Pi) was examined using malachite green as colorimetric reagent. Controls were conducted by adding the enzyme preparation after the TCA addition to correct for non-enzymatic nucleotide hydrolysis. All samples were run in triplicate, and the specific activity is reported as nmol Pi released/min/mg of protein. NTPDase was measured by spectrophotometry at 630 nm.

#### Ecto-5′-nucleotidase enzyme assay

Ecto-5′-nucleotidase/CD73 activity was determined according to previously described [36]. The reaction medium contained 10 mmol/L MgCl, 120 mmol/L NaCl, 5 mmol/L KCl, 6 mmol/L glucose and 50 mmol/L Tris–HCl buffer at pH 8.0, with a final volume of 160 μl. Intact mononuclear cells (20 µl) suspended in saline solution was added to the reaction medium (0.1 a 0.2 mg/mL of protein) and pre-incubated for 10 min at 37 °C and after the first incubation, 20 μl of AMP and a new incubation of 70 min at 37 °C was done. The reaction was initiated by the addition of substrate (AMP) at a final concentration of 2.0 mmol/L and stopped with 150 μl of 15% trichloroacetic acid (TCA). After suspending the reaction with the addition of TCA, add 200 μl of the colorimetric reagent to 20 μl of the final product. The released inorganic phosphate (Pi) was examined using malachite green as colorimetric reagent. Controls were conducted by adding the enzyme preparation after TCA addition to correct for non-enzymatic nucleotide hydrolysis. All samples were run in triplicate, and the specific activity is reported as nmol Pi released/min/mg of protein. Ecto-5′-nucleotidase activity was measured by spectrophotometry reading at 630 nm.

#### ADA enzyme assay

Adenosine deaminase activity was measured spectrophotometrically in lymphocytes [37]. Briefly, 25 μL of lymphocytes reacted with 21 mmol/L of adenosine pH 6.5 and was incubated at 37 °C for 60 min. This method is based on the direct production of ammonia when ADA acts more than adenosine. The protein content for the lymphocytes experiment was adjusted between 0.1–0.2 mg/mL. Results were expressed in U/L. One unit (1 U) of ADA is defined as the amount of enzyme required to release 1 mmol of ammonia per minute from adenosine at standard assay conditions.

#### Extracellular ATP

The quantitative ATP determination was developed using a commercial kit by bioluminescence assay with recombinant firefly luciferase and its substrate D-luciferin in serum of CM and CT group. The assay is based on luciferaseʼs requirement for ATP in producing light emission maximum ∼560 nm at pH 7.8. This assay is extremely sensitive. We combined the components of the reaction as follows to make a standard reaction solution and adjust the volumes according to requirements. Each reaction contained 1.25 μg/mL of firefly luciferase, 50 μM D-luciferin, and 1 mM DTT in 1X Reaction Buffer. After a 15 min incubation, luminescence was measured.

#### Protein quantification

Protein levels in plasma and platelets were determined by the Coomassie blue method using bovine serum albumin as standard [38]. Absorbance of samples was measured at 595 nm.

### Flow cytometry

#### NTPDase/CD39 and ecto-5’-nucleotidase/CD73 expression

Flow cytometry was utilized to evaluate the expression of ectonucleotidases NTPDase1/CD39 and ecto-5’-nucleotidase/CD73 on lymphocytes isolated from patients with CNLBP. The experimental protocol followed was performed as described previously [39]. Monoclonal antibodies specific for CD39 [anti-CD39 APC (FITC)] and CD73 [anti-CD73 PE] were employed for immunolabeling. Lymphocytes were resuspended in isotonic saline, and cell concentration was determined using a Neubauer chamber. A standardized cell counts of 1 × 10⁶ cells was used for each assay. For staining, 100 µL of the lymphocyte suspension was incubated with 20 µL of anti-CD39 and 5 µL of anti-CD73 antibodies at 4 °C for 30 min. Following incubation, the samples were centrifuged at 200 × g for 3 min at 2–8 °C, and the supernatant was discarded. The cell pellet was washed with 200 µL of saline buffer solution, and the final volume was adjusted to 200 µL before transferring to a U-bottom 96-well plate. Flow cytometric acquisition of 10,000 events per sample was performed using a BD Accuri™ C6 Plus cytometer (San Diego, CA, USA). Data analysis included assessment of size parameters (FSC), granularity (SSC) and fluorescence intensity in the relevant detection channels. Results were compared to unstained control samples and expressed as the percentage of positively stained cells. Statistical analyses were subsequently conducted to determine differences in ectonucleotidase expression.

#### Cytokines levels

Cytokines levels were assessed using commercial kits. CBA Kit (BD) (cytometric beads array) was used in this study to quantify the cytokines INFγ and TNF-α (Th1), IL-4, IL-6, and IL-10 (Th2) in the serum. Six populations of beads with different fluorescence intensities are conjugated with a specific capture antibody for each cytokine, mixed to form the CBA and read in the FL3 channel of the BD Accuri C6 Plus (BD) flow cytometer. The populations of beads were visualized according to their respective fluorescence intensities: from the least bright to the brightest. In CBA, the cytokine capture beads were mixed with the PE fluorochrome-conjugated detection antibody and then incubated with the samples forming the “sandwich” assay. The tubes for acquisition were prepared with 25 µL of sample, 25 µL of the bead mixture and 25 µL of the Th1/Th2 PE detection reagent (Human Th1/Th2 PE Detection Reagent). The same procedure was performed to obtain the standard curve. The tubes were homogenized and incubated for three hours, at room temperature, in the dark. The results were generated in graphs and tables using the CBA Analysis Software.

### Statistical analysis

The normality and homoscedasticity of data were analyzed by Shapiro–Wilk test. Data about purinergic signaling enzymes CD39/ATP, CD39/ADP, CD73/AMP, adenosine deaminase, ATP extracellular levels, CD39 and CD73 expression were analyzed by paired Student’s t-test and results were expressed as mean ± standard deviation (SD). Moreover, interleukins levels (IFN-γ, TNF-α, IL-10, IL-6, IL-4) before and after EA session were analyzed by Wilcoxon matched pairs signed-rank test. Results regarding interleukins were expressed as median ± interquartile range. To investigated if changes in inflammatory parameters were correlated with purinergic signaling modulation a Spearman Correlation was performed. Differences were considered statistically significant when p ≤ 0.05.

## Results

### Sample characterization

The study included 23 individuals, the majority of whom (60%) were women. The mean age of the participants was 46.65 ± 12.31 years. The mean duration of symptoms was 21.00 ± 13.30 weeks. Regarding the participants' pain level, they presented an average pain of 3.87 ± 1.01 based on the NPRS. Of these, 9 (39.13%) participants described the pain as mild, 12 (52.17%) moderate and 2 (8.70%) severe pain. The level of disability measured by the RMDQ was 9.609 ± 3.299. Seven participants (30.43%) were using medication to treat pain (analgesics and anti-inflammatories) and 16 (69.57%) were not undergoing any type of treatment (Table 1).

**Table 1:** Sample characterization of this study

Sample Characterization of Patients with Pain					
***Total of sample***	*Group* = 23				
***Sex***	***Nº***	***%***	***Age (years)***	***Duration of pain (weeks)***	
* Male*	9	40%	46.65 ± 12.31	21.00 ± 13.30	
* Female*	14	60%			
***Treatment to pain***	**Yes**	**No**			
	7 (30.43%)	16 (69.57%)			
***Anti-inflammatory and analgesic use***	7 (30.43%)	16 (69.57%)			
Level of disability of the participants by Roland-Morris Disability Questionnaire (RMDQ)				9.609 ± 3.299	
***Numeric Pain Rating Scale (NPRS)***				3.87 ± 1.01	
***Verbal Descriptive Pain Scale***				***Nº***	***%***
*No pain*				0	0%
*Mild pain*				9	39.13%
*Moderate pain*				12	52.17%
*Severe pain*				*2*	*8.7%*
*Very severe pain*				*0*	*0%*

### EA session increased CD73 and adenosine deaminase activity in patients with CNLBP

**Fig. 1:**
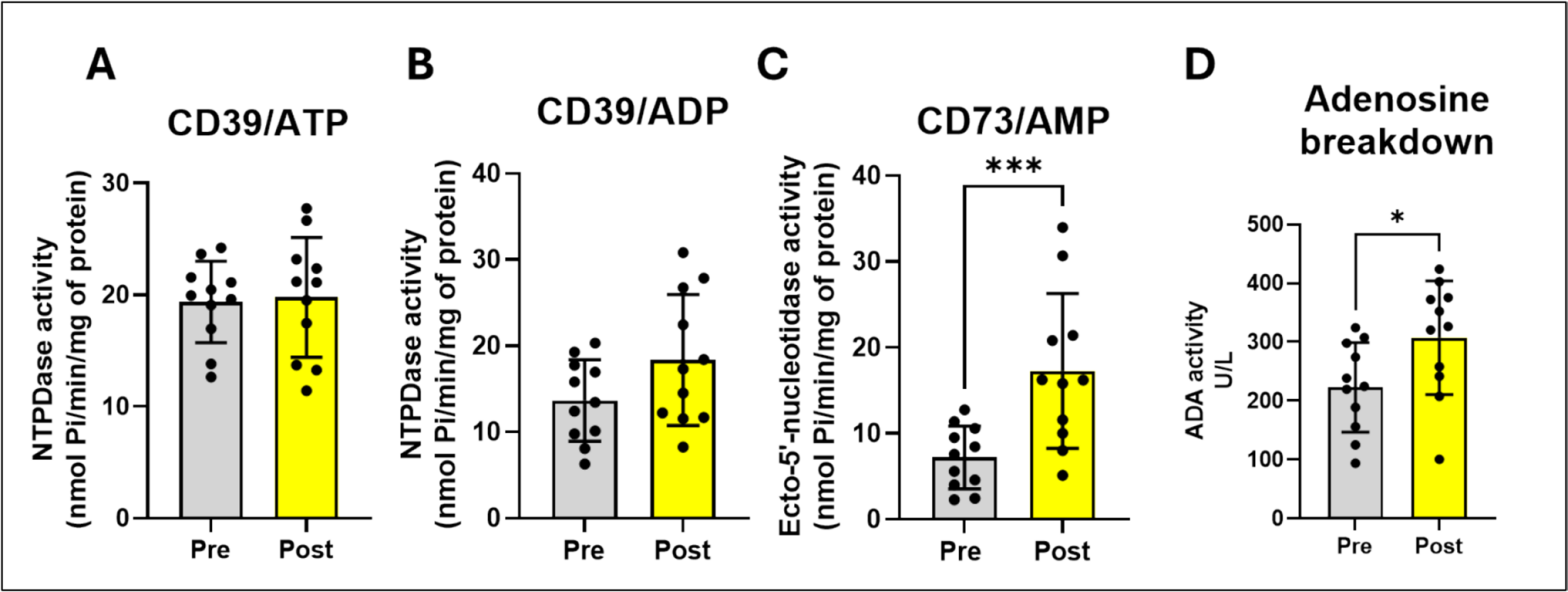
EA increases CD73 and adenosine deaminase activity in patients with CNLBP. **A** CD39 that hydrolyzes ATP to AMP (*n* = 11). **B** CD39 that hydrolyzes ADP to AMP (*n* = 11). **C** CD73 that hydrolyzes AMP to adenosine (*n* = 11). **D** Adenosine deaminase that converts adenosine to inosine (*n* = 11). Data are expressed as mean and standard deviation (SD). The results were analyzed using Student’s t-test. (*) Indicates a significant difference *p ≤ 0.05, **p ≤ 0.01, ***p ≤ 0.001 and ****p ≤ 0.0001

**Fig. 2:**
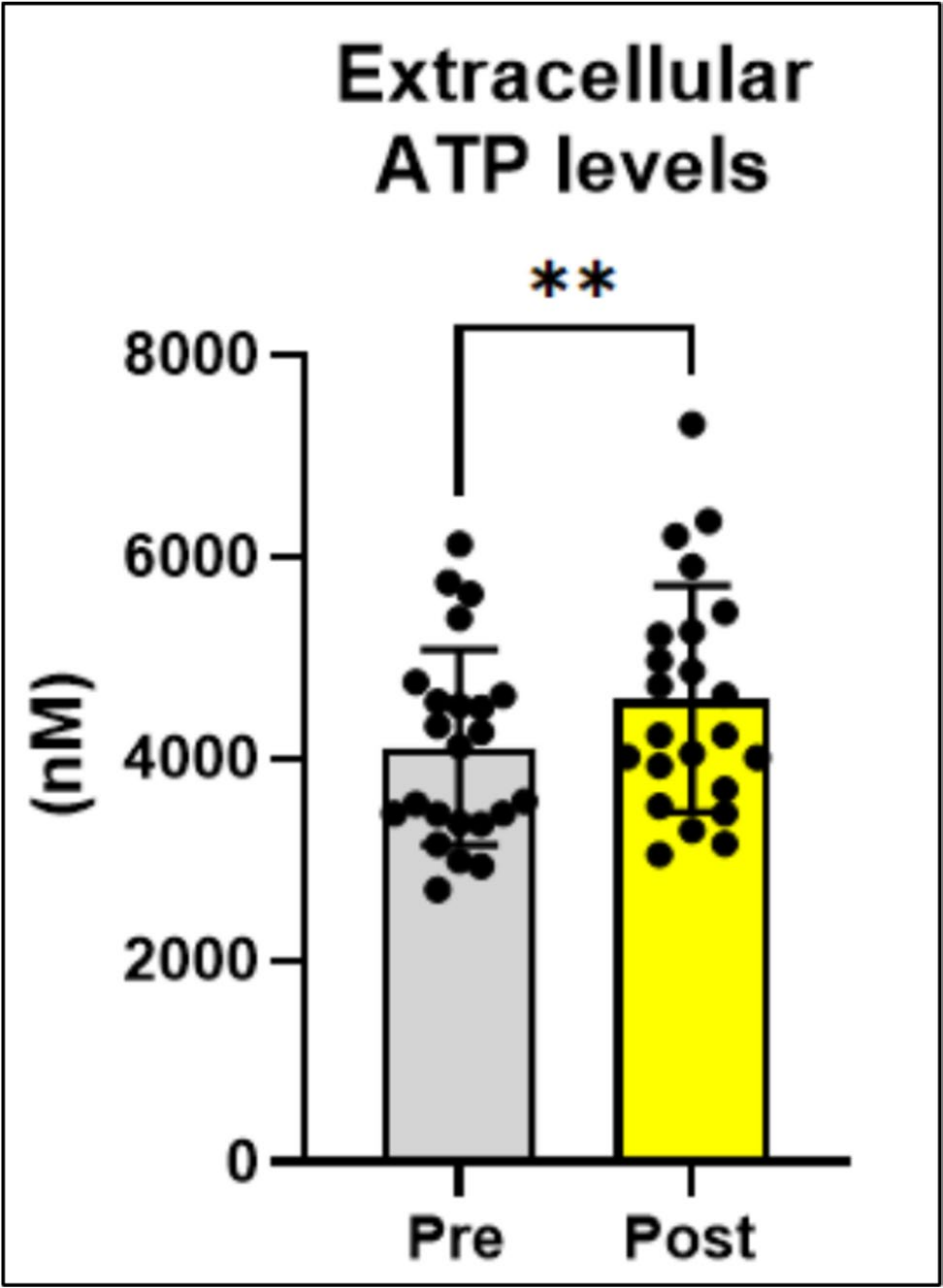
EA increases extracellular ATP levels in patients with CNLBP (*n* = 23). The results were analyzed by the Student’s t-test. (*) Indicates a significant difference *p ≤ 0.05, **p ≤ 0.01, ***p ≤ 0.001 and ****p ≤ 0.0001

Enzymes of the purinergic system can be modulated by EA protocol in patients with CNLBP. We observed an increase in NTPDase/CD39 activity in relation to ATP and ADP hydrolysis (Fig. 1A and B an increase of 2.135% respectively 34.372%), however, not significant). Nonetheless, AMP hydrolysis by ecto-5’-nucleotidase/CD73 (Fig. 1C; t_(11)_ = 5.638_,_ *p* = 0.0002), adenosine deaminase activity (Fig. 1D; t_(11)_ = 2.386_,_ *p* = 0.0382) and extracellular ATP levels (Fig. 2; t_(23)_ = 3.258_,_* p* = 0.0036) were increased after EA protocol.

### EA session increased CD73 and CD39 expression in patients with CNLBP

**Fig. 3:**
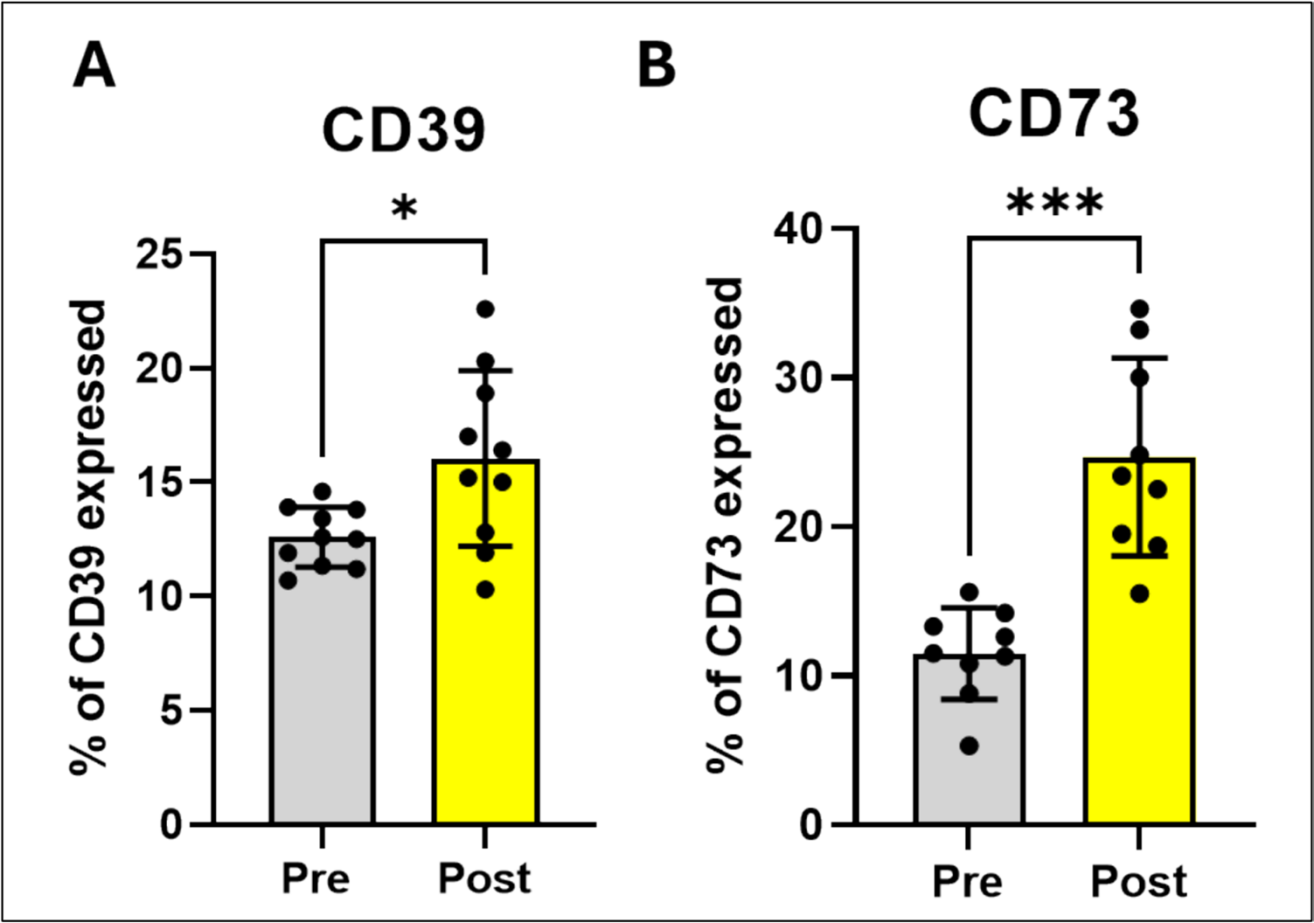
EA increases NTPDase/CD39 and ecto-5'-nucleotidase/CD73 expression in lymphocytes of patients with CNLBP. **A** NTPDase/CD39 (*n* = 10). **B** ecto-5'-nucleotidase/CD73 (*n* = 9). Data were expressed as mean and SD and results were analyzed by the Student’s t-test. (*) Indicates a significant difference *p ≤ 0.05, **p ≤ 0.01, ***p ≤ 0.001 and ****p ≤ 0.0001

In addition, expression of the NTPDase/CD39 (Fig. 3A; t_(10)_ = 2.60, *p* = 0.0287) and ecto-5'-nucleotidase/CD73 (Fig. 3B; t_(10)_ = 5.33, *p* = 0.0007) in lymphocytes of the patients with CNLBP was increased after the EA session.

#### Cytokines levels are modulated by one section of the EA

**Fig. 4:**
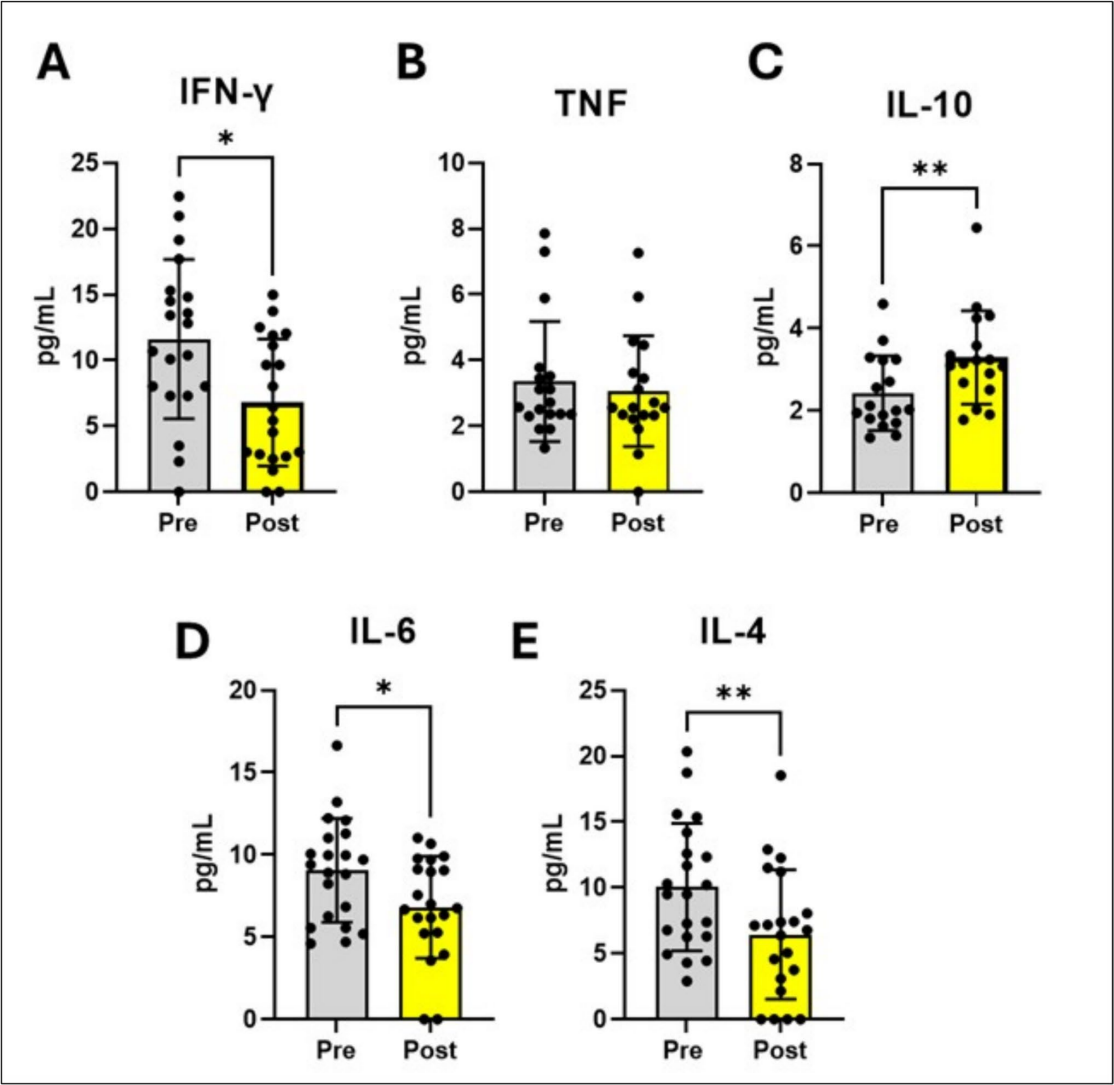
EA modulates pro and anti-inflammatory cytokines levels in the patients with CNLBP. **A** IFN-y (*n* = 20). **B** TNF-α (*n* = 18). **C** IL-10 (*n* = 17). **D** IL-6 (*n* = 21). **E** IL-4 (*n* = 21). Data were expressed as mean and SD or median and interquartile range. The results were analyzed by Wilcoxon matched signed-rank test. (*) Indicates a significant difference *p ≤ 0.05, **p ≤ 0.01, ***p ≤ 0.001 and ****p ≤ 0.0001

Inflammatory interleukin levels in the serum of patients with CNLBP were analyzed before and after EA protocol. A decrease in INF-γ (Fig. 4A; W = −124.0; *p* = 0.0192), IL-6 (Fig. 4D; W = −134.0; *p* = 0.018), IL-4 Fig. 4E; W = 119.0, *p* = 0.0031) was observed after the EA session. On the other hand, anti-inflammatory IL-10 was increased after the EA session (Fig. 4C; W = 119.0; *p* = 0.0031) and TNF-α levels were not changed by the EA session (Fig. 4B W = −27.00; *p* = 0.5477). To investigate the relationship between purinergic signaling and inflammatory responses, correlations between purinergic signaling markers and pro-and anti-inflammatory interleukins were performed (Table 2). Strong negative correlations were observed before EA session between CD39 enzyme that hydrolyzes ATP and TNF-α (*r* = −0.662, *p* = 0.030), adenosine deaminase and TNF-α (*r* = −0.812, *p* = 0.003). Furthermore, after EA session, strong negative correlations were identified between CD73 expression and TNF-α levels (*r* = −0.786, *p* = 0.015), CD39 enzyme that hydrolyzes ADP and IL-4 (*r* = −0.690, *p* = 0.022), adenosine deaminase and IL-6 (*r* = −0.683, *p* = 0.020).

**Table 2:** Correlations between purinergic signaling and inflammatory parameters

Purinergic signaling markers	Inflammatory parameters									
	**IFN-γ**		**TNF-α**		**IL-10**		**IL-6**		**IL-4**	
**Before EA**	r	*p*	r	*p*	r	*p*	r	*p*	r	*p*
CD39 expression	−0.431	0.212	0.140	0.699	−0.067	0.862	0.224	0.536	0.151	0.682
CD73 expression	0.050	0.911	0.420	0.261	0.305	0.427	0.133	0.743	0.550	0.132
ATP extracellular	−0.224	0.302	0.055	0.800	0.113	0.605	−0.226	0.298	0.147	0.500
CD39/ATP enzyme	−0.205	0.542	**−0.662**	**0.030**	−0.232	0.487	−0.045	0.903	−0.609	0.051
CD39/ADP enzyme	0.150	0.657	−0.137	0.687	0.176	0.599	0.000	0.999	−0.272	0.418
CD73 enzyme	0.492	0.126	0.0958	0.779	−0.307	0.354	0.118	0.734	0.190	0.576
Adenosine deaminase	0.086	0.801	**−0.812**	**0.003**	−0.595	0.058	−0.190	0.576	−0.263	0.434
	**IFN-γ**		**TNF-α**		**IL-10**		**IL-6**		**IL-4**	
**After EA**	r	*p*	r	*p*	r	*p*	r	*p*	r	*p*
CD39 expression	−0.063	0.853	−0.437	0.179	−0.446	0.168	0.018	0.961	−0.400	0.225
CD73 expression	0.225	0.558	**−0.786**	**0.015**	0.220	0.573	−0.108	0.783	0.066	0.880
ATP extracellular	−0.160	0.463	0.023	0.915	−0.027	0.901	−0.155	0.479	−0.014	0.947
CD39/ATP enzyme	0.391	0.232	0.355	0.281	−0.241	0.469	0.259	0.438	−0.600	0.056
CD39/ADP enzyme	0.086	0.801	−0.036	0.918	−0.200	0.551	−0.100	0.769	**−0.690**	**0.022**
CD73 enzyme	0.496	0.122	0.638	0.052	0.093	0.785	−0.277	0.405	−0.518	0.107
Adenosine deaminase	−0.091	0.790	0.255	0.445	0.525	0.099	**−0.683**	**0.020**	0.354	0.286

Data are analyzed by Spearman correlation test and significant values (p < 0.05) are marked in bold. Spearman’s correlation coefficient (r) and p values are shown. CD39 expression (*n* = 10), CD73 expression (*n* = 9), ATP extracellular (*n* = 23), CD39/ATP (*n* = 11), CD39/ADP (*n* = 11), CD73/AMP (*n* = 11), adenosine deaminase (*n* = 11). IFN-y (*n* = 20), TNF-α (*n* = 18), IL-10 (*n* = 17), IL-6 (*n* = 21), IL-4 (*n* = 21)

## Discussion

CNLBP is considered the leading cause of disability worldwide, with an increase, especially in low- and middle-income countries. Treatment varies from person to person, as not all patients respond to the same therapeutic approach. In these patients, treatment aims to reduce symptoms and disability, allowing a return to activities of daily living [4]. An alternative to alleviate the symptoms of CNLBP is treatment with EA, which has been recommended as a non-invasive and multitarget therapy [40]. In our study, we observed that a single 20-min EA session was able to enhance purinergic signaling and reduce the levels of interleukins secreted by immune cells. In the following section, we will discuss in more detail the molecular effects of EA in patients with CNLBP.

### EA modulates purinergic signaling in patients with CNLBP

ATP extracellular and its hydrolyzed products play important roles in pain transmission. Our results show ATP extracellular levels increased in patients with CNLBP after EA protocol. We believe that increased ATP extracellular levels have been the key to the pathway of effectiveness of EA treatment. Different studies have demonstrated that release of ATP by EA has a relationship with analgesic and anti-inflammatory symptoms [22, 41–43]. Probability of the ATP extracellular levels be used as a biomarker for evaluating EA effects of the patients in treatment. However, increased ATP levels are paradoxical, in general, situations with higher levels are associated with inflammatory responses. Because the purinergic system does not degrade/detoxify environments with higher ATP amounts [44]. Thus, ATP in large quantities in the extracellular space is a signaling molecule for the sensation of harmful stimuli in the peripheral system [45]. That way, we believe in the hypothesis/paradoxical that high levels of ATP have indirect analgesic and anti-inflammatory effects/actions through the products of its degradation. So, it is necessary to release ATP by EA to ameliorate pain symptoms by hydrolysis ATP pathway enzymes of the purinergic system.

Complex biochemical engineering is required for the hydrolysis of ATP and the generation of its products. The purinergic pathway is composed of several purine-hydrolyzing enzymes expressed on the cell surface, known as ectoenzymes. These enzymes include NTPDase/CD39, which hydrolyzes ATP and ADP in ADP and AMP respectively, and ecto-5'-nucleotidase/CD73 that hydrolyzes AMP at adenosine. In addition, adenosine deaminase (ADA) is also present and acts converting nucleoside adenosine (ADO) to inosine [39, 46]. Although EA protocol did not change NTPDase/CD39 activity, we observed that CD39 expression in lymphocytes significantly increased after EA. We suggest that this result should be correlated with higher extracellular ATP levels present after EA protocol. Although NTPDase activity did not significantly change following EA, an increase of 30% in NTPDase/CD39 that hydrolyzes ADP into AMP) was observed suggesting a trend toward a modulation of EA on CD39 activity. Perhaps one single EA session was insufficient to significantly increase this enzyme activity. Nonetheless, both ecto-5’-nucleotidase/CD73 expression and activity were significantly increased after EA protocol, suggesting a large amount of adenosine being produced. This finding is compatible with increases in ADA activity observed after EA protocol. Interestingly, ATP released in response to mechanical stimulation through EA should bind to purinergic receptors (*e.g.* P2X and P2Y) [21]. Overall, ATP can be also rapidly degraded into ADO by extracellular ectonucleotidases. The increased ADO levels of the organisms exert antinociceptive and anti-inflammatory effects and decrease feeling pain [42, 47]. ADO is a nucleoside and neuromodulator responsible for regulating different processes through the activation of G-protein-coupled receptors (adenosine receptors). ADO and its receptors are widespread in the central and peripheral nervous system and play an important role in the processing of nociceptive sensory signals in different pain models [48]. In addition, ADO receptors are highly expressed in lymphocytes on CD4 + T cells [49]. Thus, noninvasive stimulation via EA should generate ADO-mediated antinociceptive effects via adenosine A_1_ receptors [22]. Furthermore, EA can increase the expression of adenosine A_3_ receptors, ADO, and immune-related factors, which promote proliferation and anti-inflammatory effects in fibroblasts [11]. In general, our findings yielded that a single EA session modulates purinergic signaling and expression in lymphocytes samples and we suggest that these changes should be related with both pro and anti-inflammatory actions.

### Pro and anti-inflammatory interleukin levels are altered by the EA protocol

To establish a link between purinergic signaling and inflammation in patients with CNLBP, we performed correlations between purinergic signaling endpoints and inflammatory cytokines, supporting the influence of purinergic signaling on immune activation resulting in anti-inflammatory responses observed. There is a close relationship between the purinergic signaling and immunologic system. Higher extracellular levels of ATP and ADP have been shown to be associated with pro-inflammatory effects due to the production of IL-6, TNF-α [50, 51]. However, the effects of elevated ADO levels, resulting from the hydrolysis of ATP and ADP, are anti-inflammatory reducing the production of pro-inflammatory cytokines [50, 52]. Our results show a decrease in pro-inflammatory interleukins such as INF-γ, IL-4, IL-6 and increase anti-inflammatory levels IL-10 after EA intervention on patients with CNLBP. IL-10 is a cytokine with anti-inflammatory and analgesic effects [53, 54]. The effects produced by IL-10 are directly associated with the inhibition of nociceptive activity and indirectly with the decrease in edema formation by decreasing the production of pro-inflammatory cytokines by defense cells [55, 56]. In previous studies in IL-10 knockout mice, increased nociceptive behavior was observed. It was also observed that in untreated patients with low back pain, IL-10 levels were decreased and the levels of the pro-inflammatory biomarkers C reactive protein, IL-6, and TNF-α were increased [57–59]. Furthermore, EA treatment significantly increased the threshold of mechanical and heat nociception. This effect was related to an increase in IL-10 levels in paw and spinal cord tissues. Thus, it is believed that the analgesic and anti-inflammatory effect of EA treatment may be partly associated with the suppression of pro-inflammatory cytokines mediated by IL-10 induction [60, 61]. IL-10 plays an essential role in reducing pain sensitivity and inflammation progression, making it an interesting target for pain control.

Previous studies have demonstrated a relationship between products generated by the purinergic system and increased levels of IL-10. An example of this is the activation of adenosine receptors (A_2a_ and A_2b_) through high levels of extracellular ADO, which inhibit the release of TNF-α, IL-6, and IL-4, in addition to increasing the release of IL-10 [18, 23, 51, 62, 63]. Furthermore, high levels of AMP can inhibit the release and increase the levels of IL-4, TNF-α, and IFN-γ [23, 64]. In contrast, reduced levels of IL-4 are associated with pain relief and inflammation control. Together our findings were supported by the strong negative correlations observed to adenosine deaminase and IL-6 levels and CD73 expression and TNF-α levels, CD39/ADP and IL-4 levels, suggesting that purinergic signaling after EA session promotes anti-inflammatory responses in patients with CNLBP.

Additionally, ADO generated in the organism can activate transmembrane purinergic P1 receptors (A_1_, A_2A_, A_2B_, and A_3_), which are known to be G protein–coupled receptors (GPCRs) [51]. It is currently understood that ADO activates A_1_ and A_3_ receptors to regulate the activity of immune cells such as monocytes, macrophages, and leukocytes. However, the specific and functional roles of A_1_ and A_3_ receptors when activated by ADO within the tissue microenvironment remain unclear [49–51]. The immune system is associated with the promotion of inflammation, which may be modulated by the anti-inflammatory effects of EA [12, 13]. The literature also suggests that EA may exert antinociceptive effects in pain control through A1 receptor–mediated mechanisms [22]. Moreover, transmembrane A_2A_ and A_2B_ receptors, when activated by ADO in the extracellular microenvironment, can inhibit the release of TNF-α, IL-6, and IL-4, while increasing IL-10 levels [18, 23, 51, 62, 63]. In this context, the present study demonstrated that IL-6 and IL-4 levels were reduced in participants following EA, whereas IL-10 levels were increased after EA. These findings suggest that a single session of EA may exert significant effects on purinergic signaling and on the regulation of inflammatory mediators associated with the treatment of CNLBP.

**Fig. 5:**
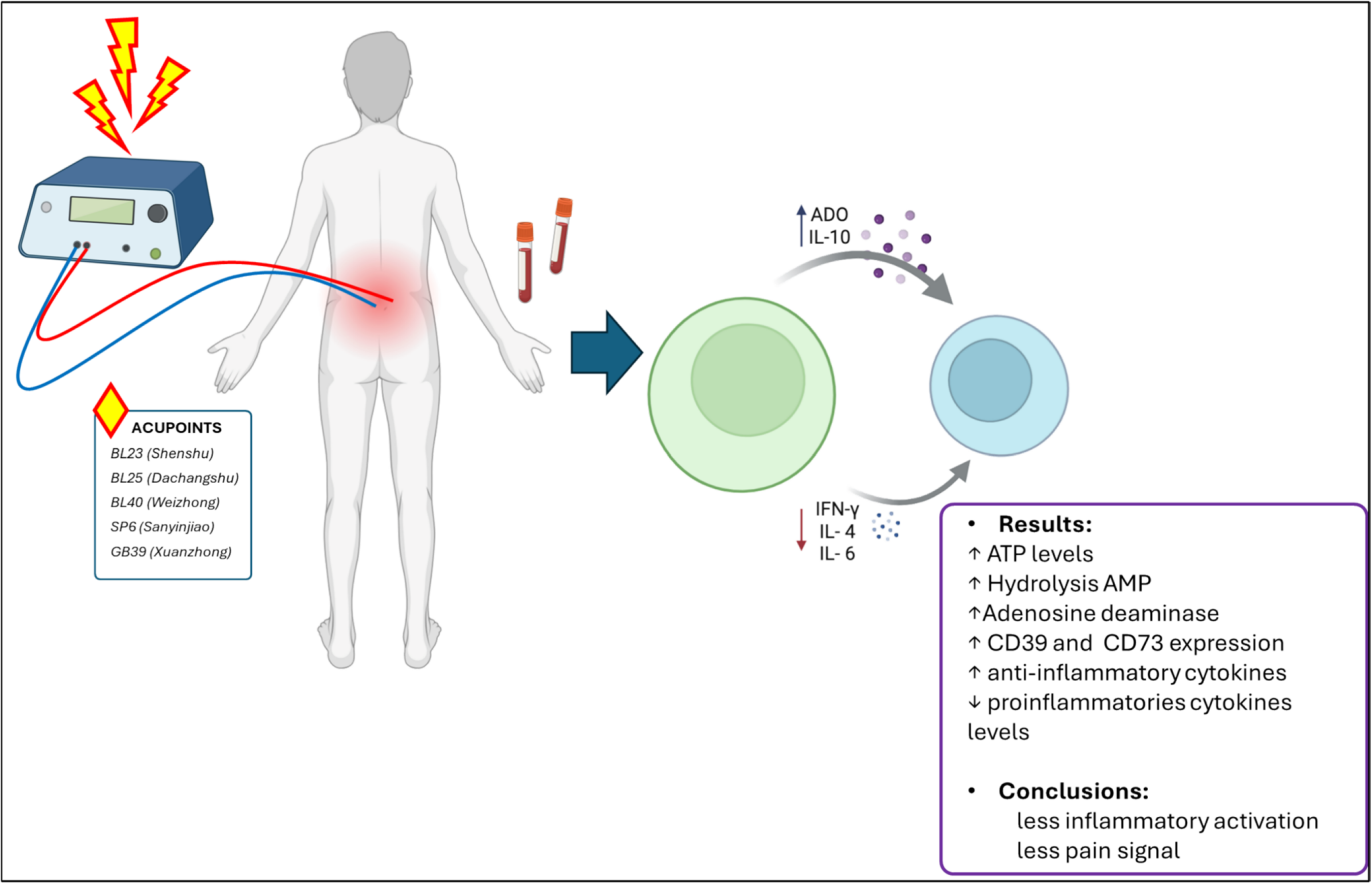
A single EA session promotes anti-inflammatory responses by modulating purinergic signaling

In this study, we investigated the effects of a single session of EA in patients with CNLBP. Overall, our findings suggest that EA elicits purinergic signaling and anti-inflammatory responses which may contribute to its analgesic effects (Fig. 5). However, we did not assess pain thresholds following the EA protocol in this study. In addition, the EA effects were analyzed 30 min after intervention that is considered a short time window to understand long time clinical responses. Further research is needed to better understand the effects of repeated EA sessions in patients with chronic pain.

### Limitations

In addition to the previously mentioned limitations namely the small sample size, the single-session intervention, and the short observation period we now explicitly acknowledge several other important constraints. First, a major limitation of this study is the absence of a control group, which limits causal inference. Without a comparator, confounding factors such as placebo effects and temporal variations cannot be excluded. Therefore, the findings should be interpreted with caution, and randomized controlled studies are needed to confirm these results. Second, the study focused primarily on biochemical and molecular parameters related to inflammation and purinergic signaling and therefore did not evaluate broader clinical outcomes such as long-term symptom improvement, functional recovery, or quality of life measures.

It is important to emphasize that, although Th1 (IFN-γ, TNF-α) and Th2 (IL-4, IL-6, IL-10) cytokines were investigated, the analysis did not include other pro-inflammatory cytokines, such as IL-1β, IL-2, and interferon-γ, nor other inflammatory mediators, including C-reactive protein, all of which are known to play a role in the regulation of chronic pain, as reported in the literature [15, 16]. This limitation may hinder a comprehensive understanding of the effects of EA on inflammatory markers in patients with non-specific chronic low back pain. Therefore, further studies evaluating a broader range of cytokines and inflammatory mediators are recommended to better characterize the inflammatory microenvironment in patients with non-specific chronic low back pain. Furthermore, the results did not demonstrate statistically significant differences in TNF-α levels before and 30 min after a single EA session. Thus, additional studies are needed to clarify whether a single EA session is insufficient to modulate TNF-α levels, or whether the assessment time should be extended beyond the 30-min interval standardized in the present study.

Furthermore, we recognize that potential confounding factors may have influenced the results. Although participants were screened according to the study criteria, variables such as concomitant medications, ongoing treatments, lifestyle factors, and individual physiological variability were not fully controlled and may have affected the observed responses. These aspects are now discussed in the revised manuscript as additional limitations. Therefore, future studies should investigate the temporal dynamics of EA-induced biochemical responses by including multiple treatment sessions and different follow-up time points. Longitudinal analyses assessing the purinergic system and inflammatory cytokines after repeated EA interventions may help clarify whether the acute molecular changes observed in the present study translate into sustained physiological and clinical effects. Such approaches could provide a better understanding of the persistence, cumulative regulation, and therapeutic relevance of EA-mediated modulation of purinergic signaling in patients with chronic nonspecific low back pain.

## Conclusion

CNLBP is one of the most prevalent and disabling conditions globally, representing the leading cause of physical disability. Non-invasive therapies such as EA have emerged as promising alternatives for pain management. We demonstrated that a single 20-min session of EA was sufficient to acutely modulate key components of pain and inflammatory signaling. Specifically, EA increased the expression of NTPDase/CD39 and ecto-5'-nucleotidase/CD73, enhanced AMP hydrolysis, ADO activity and extracellular ATP levels. These changes were accompanied by a decrease in pro-inflammatory markers, including IL-4, IL-6, and INF-γ, and an increase in the anti-inflammatory cytokine IL-10. Correlation analysis suggests that EA modulates the purinergic signaling pathway and cytokine balance, contributing to its beneficial effects. These results provide mechanistic insights into the anti-inflammatory properties of EA and support its potential as a complementary treatment for CNLBP. However, further studies are needed to better understand the acute and chronic effects of EA in patients with pain.

## Data Availability

No datasets were generated or analysed during the current study.
